# Competition between radiative and predissociative decay mechanisms in excited electronic states of CH radical

**DOI:** 10.1039/d5cp04531b

**Published:** 2026-03-06

**Authors:** Andrei Sokolov, Sergei N. Yurchenko, Jonathan Tennyson

**Affiliations:** a Department of Physics and Astronomy, University College London Gower St London WC1E 6BT UK j.tennyson@ucl.ac.uk

## Abstract

The CH radical is an important astrophysical molecule due to its ubiquity in the universe, and its relatively low dissociation threshold leads to manifestations of photodissociation and predissociative behaviour at low photon energies. This study investigates the competition between radiative decay and predissociation in three excited electronic states of the CH radical, A ^2^Δ, B ^2^Σ^−^, and C ^2^Σ^+^. The predissociation processes are modelled using a combination of the stabilization approach based on rigorous solutions of the Shrödinger equation using code Duo, and WKB-type approach using code LEVEL to describe Feshbach and tunnelling resonances, respectively. Total lifetimes compare favourably with experimental data for A ^2^Δ and C ^2^Σ^+^ states, but our B ^2^Σ^−^ predictions are understimated above the dissociation threshold. The predissociative lifetimes for levels belonging to A ^2^Δ and C ^2^Σ^+^ states are often found to be comparable to their radiative lifetimes. These weak predissociation effects in A ^2^Δ and C ^2^Σ^+^ states and coupling mechanisms responsible for them are discussed.

## Abstract 2

Levels in excited electronic states with weak couplings and long radiative lifetimes can show weak predissociation, and molecules in those states are as likely to emit a photon as to break apart.

## Graphical abstract

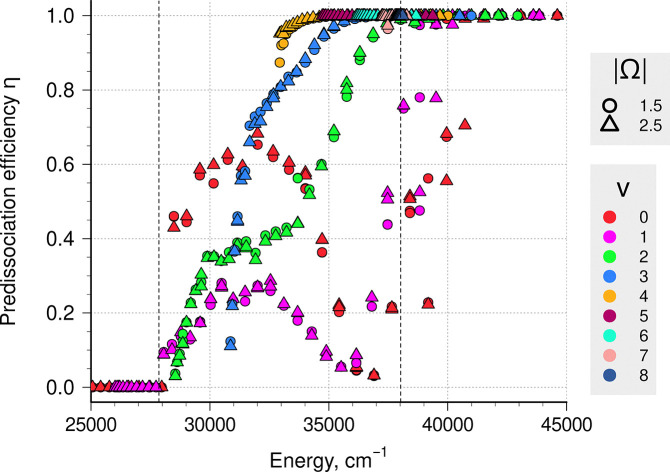

## 1 Introduction

The methylidyne radical or CH is a well-studied species with a number of applications, including fundamental physics,^1^ combustion,^2^ and in particular astrophysics.^3,4^ This molecular species has a relatively low dissociation threshold (∼3.6 eV) and can thus dissociate into individual atoms by absorbing a low energy photon and getting excited into the state continuum. Alternatively, CH molecules can get promoted into quasi-bound states, states with a largely bound character located above the dissociation threshold. In this work, we focus on these excited states and, in particular, treating the competition between their decay by spontaneous emission *versus* predissociation, a radiationless process, where a molecule in a quasi-bound state breaks apart.^5^ Account of predissociation is important for accurate interpretation of spectra,^6^ as well as a potential contributor to various chemical networks.^7^

In scattering theory, the quasi-bound states of diatomics show as either Feshbach or shape resonances depending on whether the bound and continuum wavefunctions in the uncoupled solution belong to different electronic states or the same one.^8^ Herzberg^9^ labels these as case I and case III predisociation processes, respectively. Several theoretical approaches can describe these in a time-independent manner; see Uhlíková *et al.*,^10^ Császár *et al.*,^11^ Mussa and Tennyson^12^ and Silva and co-workers.^13,14^ Detailed treatment of predissociation in the lowest few electronically excited states is particularly important, as radiative and predissociation rates (or lifetimes) in this energy range can have comparable magnitudes, while for highly excited states, which usually undergo a number of curve crossing, predissociation most often dominates the total rate. For diatomics, the electronic transitions into these low-lying electronic states are often the strongest spectral features;^15^ this is also the case for CH.

Obtaining (state-resolved) predissociation efficiencies, defined as a fraction of predissociation rate to the total rate, are essential for certain astrophysical applications^16^ and elsewhere. In this work, we focus on the methodology for obtaining predissociation efficiency, *η*, total lifetimes, and dissociation rates *k*_pred_ for the A ^2^Δ, B ^2^Σ^−^ and C ^2^Σ^+^ electronically excited states of CH. Below, in Section 2 we describe the electronic structure data, our treatment of both electronic and rotational predissociation, and introduce the concept of predissociation efficiency. In Section 3 we discuss the results and comparisons with the available data for each electronic state. Conclusions and plans for future work are given in Section 4.

## 2 Methodology

### 2.1 Electronic structure data

The electronic structure data used in this work come from a recent paper by Hou and Liu,^17^ who provided an extensive set of potential energy curves (PECs), permanent and transition dipole moments (PDMs, TDMs), spin–orbit couplings, and electronic coupling curves for the CH radical.

Minor changes have been introduced in the adiabatic curves of the six lower electronic states (X ^2^Π, a ^4^Σ^−^, A ^2^Δ, B ^2^Σ^−^, C ^2^Σ^+^, b ^4^Π) to ensure correct asymptotic behaviour. The dissociation energy was set to *D*_e_ = 29 301.07 cm^−1^, and with the zero-point energy from the DUO calculation of ZPE = 1401.42 cm^−1^, one gets *D*_0_ = 27 899.65 cm^−1^. This value is consistent with the experimental estimates of *D*^expt^_0_ = 27 856.34 ± 100 cm^−1^ (ref. 18) and 27 950 ± 80 cm^−1^,^19^ but not with 27 960 ± 10 cm^−1^.^20^ The second dissociation limit to which the A ^2^Δ and C ^2^Σ^+^ states tend to, was obtained by adding ^1^D term value of the carbon atom^21^ to *D*_e_.

The tails of the *ab initio* PECs were fitted to^22,23^
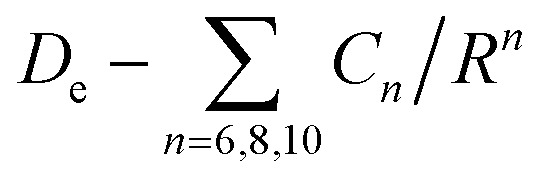
based on their behaviour around 3 Å and then spliced with the rest of the curve using a sharp sigmoid. Stwalley^22^ warns against extrapolating from too short a distance, where “chemical” (as opposed to long-ranged) forces dominate, as this could lead to incorrect values of *C*_*n*_ coefficients. From an estimate based on an expression by Le Roy,^22^ such an extrapolation is valid for *R* > 2(〈*r*_C_^2^〉^1/2^ + 〈*r*_H_^2^〉^1/2^), where 〈*r*^2^〉 represents the mean square radius of the outermost electron orbital. Substituting the values from Lu *et al.*,^24^ one obtains *R* > 3.9 Å. We chose a shorter distance because at 3.9 Å the *ab initio* X ^2^Π and B ^2^Σ^−^ points are already above the target asymptotic *D*_e_ value. While more robust and physically informed ways to construct long-ranged potentials for open-shell diatomics exist,^25^ the present scheme is simpler and ensures a good position for the lowest (discretized) unbound state and the barrier height.

These PECs were used as inputs in the DUO code,^26^ which then performs nuclear motion calculations by variationally solving the rovibronic Shrödinger equation for *N* coupled electronic states. Recently, a method to calculate predissociation lifetimes due to curve crossing with the help of DUO has been developed in our group. It relies on the well-established stabilization approach.^27,28^ For full details, see Mitev *et al.*^29^ and Uhlíková *et al.*^10^ Here, we only provide a brief description of the approach and then focus on the new features.

### 2.2 Treatment of predissociation

To solve the Shrödinger equation, DUO imposes boundary conditions at long range by the truncation of the grid at *R*_max_. If *R*_max_ is set large enough for the given potential, it has no impact on the true bound states where the wavefunctions decay exponentially outside their potential wells. At the same time, the continuum energy spectrum gets discretized, which allows us to obtain the continuum wavefunctions obeying the boundary conditions for a particular *R*_max_ and differing from scattering solutions only by a normalization factor.^30^ If a bound solution is coupled to the continuum, its computed eigenvalues will experience fluctuations as a function of *R*_max_ due to perturbation by different continuum states, making the state quasi-bound.^28^

For successive Hamiltonian diagonalizations at various *R*_max_, the density of states for each quasi-bound level “stabilizes” around the true, *R*-independent value. The density of states can be linked to the scattering matrix,^31,32^ and in simple cases, it can be approximated by a sum of Lorentzians for each individual resonance,1
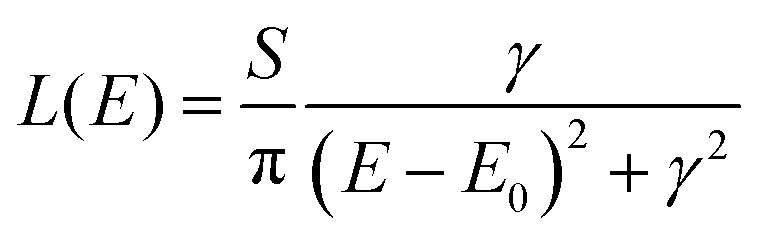
where *γ* is half-width at half-maximum (HWHM), *E*_0_ is the true energy of the quasi-bound level, and *S* is a scaling factor equal to 1 for the density. With a large enough sample size, we can approximate the density of states directly by binning the energies of the quasi-bound states obtained.

Fig. 2 shows an example of such a quasi-bound state. The plot of eigenenergy of a state as a function of *R*_max_ (upper panel) shows clustering around the true energy of this quasi-bound level, and the widths of these distributions (lower panel) can be used to calculate rates and lifetimes*τ*_case I_ = 1/*k*_case I_ = 1/(4π*cγ*).

The histograms are generated with a fixed number of bins (100–200) per resonance, unlike in Uhlíková *et al.*,^10^ where a constant bin width Δ*E* = 0.5 cm^−1^ was used. Our choice of binning allows one to resolve peaks for both very narrow and wide resonances. To obtain the respective Lorentzians, we also test a different fitting procedure from that described in Mitev *et al.*^29^ The resonance shapes are obtained using the weighted linear least squares fit (wLSF) as
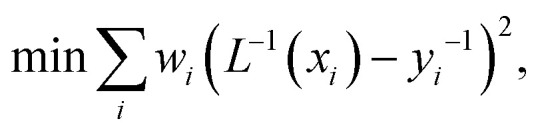
where *L* is the Lorentzian distribution with parameters (*S*,*γ*,*E*_0_) for each resonance. While *L*(*x*) is a non-linear function of *x*, its inverse is quadratic *L*^−1^(*x*) = *a*_2_*x*^2^ + *a*_1_*x* + *a*_0_, so the usual LSF techniques can be used to get the coefficients and map them back (*a*_2_,*a*_1_,*a*_0_) → (*S*,*γ*,*E*_0_). The weights *w*_*i*_ are needed to prioritize the core of the Lorentzian as opposed to the large noisy wings in *L*^−1^(*x*), and are chosen as *w*_*i*_ = *L*(*x*_*i*_)^*n*^, where *n* is some positive power. This power is optimized separately with a non-linear algorithm: after setting *n*_init_ = 6, a wLSF is made for the inverse Lorentzian profile to the inverse of the data as shown above. This procedure is repeated for different values of *n* until the root mean squared deviation is minimized. The fitting procedure is quick, robust, and does not requires initial estimates for the Lorentzian parameters, which is why it can be applied in semi-automatic fashion.

The approach described above relies on quantum number assignments to track stabilization of specific (quasi-bound) states from the entire set of solutions (compare Fig. 2 with Uhlíková *et al.*,^10^ Fig. 10). However, the assignments for approximate quantum numbers (*i.e.*, *v*, *Ω*) are not always reliable or consistent across calculations with varying *R*_max_. When the number of misassignments is small, it is possible to treat them as outliers in the fitting procedure, but this approach fails for states above the respective dissociation limit of the potential.

To be able to describe predissociation lifetimes in this range of energies, we also have to consider shape (case III) resonances. We attack these problematic states by treating case I and case III predissociation differently. Rotational predissociation is treated using code LEVEL,^33^ which produces resonance widths for a given PEC that can be converted to rates and lifetimes as*τ*_case III_ = 1/*k*_case III_ = 1/(2π*cγ*_LEVEL_).

We assume that predissociation rates due to tunnelling quickly become the dominant mechanism for dissociation, which is why it is safe to keep case I rates constant. The constant value is taken to be that of the last successful stabilization result within the group (*J*,*Ω*,parity). Thus, the total dissociation rate above the threshold should be well approximated by *k*_(*J*,*Ω*,*e*/*f*) threshold_ + *k*_case III_. The two mechanisms are independent so there is no double counting.

A caveat with this mixed treatment is that unlike DUO, LEVEL does not support couplings between electronic states, so even using the same set of PECs as in our DUO calculations does not guarantee consistent energies or even number of levels for the same electronic state between the two calculations. Regardless of that, we use *τ*_case III_ values from LEVEL if a state is present in both codes, and if DUO predicts more predissociative states, an arbitrarily small value (1 fs) is set as their lifetimes. The assignment of rotational quantum numbers *N* and *F*_*i*_ to the DUO results, which is needed for merging with LEVEL output, was performed following the standard conventions:^34,35^ for *J* ≥ *Λ* + *S*, the spin substates *F*_1_,…,*F*_2*S*+1_ within each group of (state, *v*, *J*) were assigned in order of increasing energy, and the values of *N* were assigned from *N* = *J* − *S* to *N* = *J* + *S* in the same order.

In summary, for levels experiencing case I predissociation, we use DUO to obtain level energies for a large number of box sizes and retrieve the predissociative widths of the quasi-bound levels from their state densities. Above the respective dissociation limits, we assume *τ*_case I_ stays constant, and we use LEVEL to estimate the *τ*_case III_ lifetimes. The rates for the two processes yield the total predissociation rate.

**Fig. 1:**
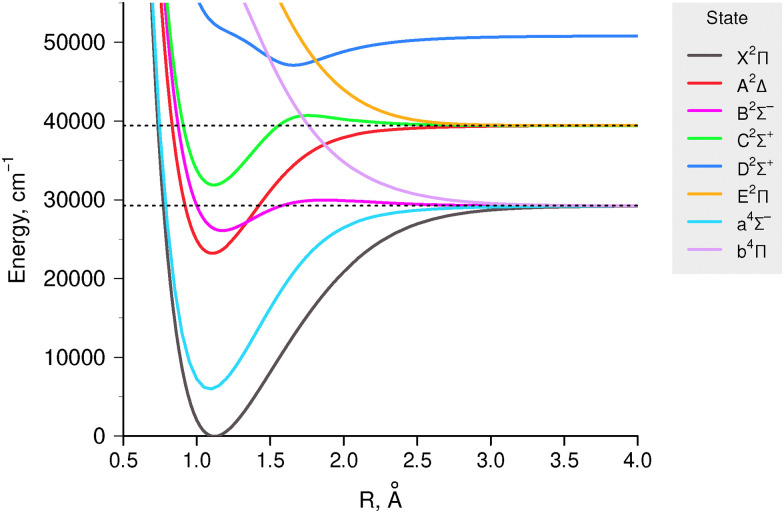
Lowest potential energy curves of the CH radical. Dashed lines represent the first and second dissociation limits, which we focus on.

Here, we focus on predissociation in three excited electronic states of the CH radical, namely A ^2^Δ, B ^2^Σ^−^, and C ^2^Σ^+^ (see Fig. 1). The B ^2^Σ^−^ state goes to the first dissociation limit and can only exhibit predissociation due to tunnelling through the potential barrier. At the same time, A ^2^Δ and C ^2^Σ^+^ PECs correlate with the second dissociation limit and their wavefunctions are immersed in the continuum of underlying states, so both case I and III predissociation mechanisms are possible.

**Fig. 2:**
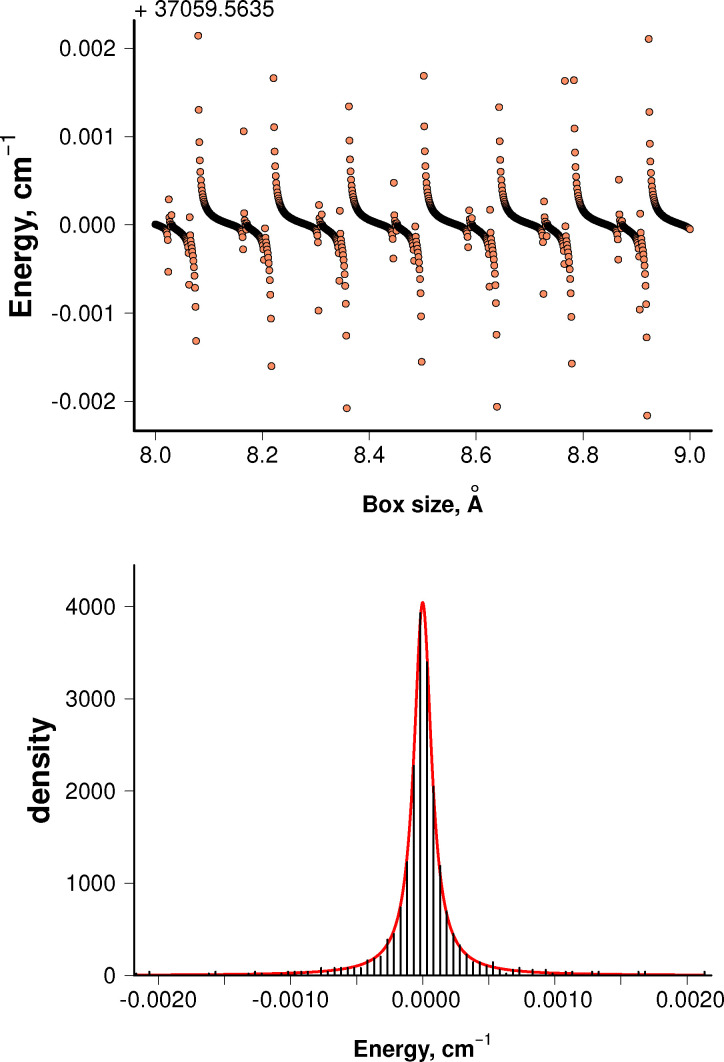
Example of the energy stabilization for the *v* = 0, *J* = 18.5, *Ω* = −0.5 quasi-bound level of the C ^2^Σ^+^ state (upper panel) and the respective energy density distribution with an overlaid fitted Lorentzian curve (lower panel) with parameters *E*_0_ = 37 059.563543 cm^−1^, *γ* = 8.087 × 10^−5^ cm^−1^, and *S* = 1.028. The quality of the fit is representative for the vast majority of case I resonances considered in this work.

For the case I calculations, the position of the right-side wall of the simulation box is incrementally shifted from 8 to 8.999 Å in steps of 0.001 Å, while also maintaining the uniform grid of the sinc-DVR basis functions.^36^ The small reduced mass of CH leads to relatively wide spacing between the unbound vibrational levels,^10^ so we choose a large *R*_max_ value to compress the vibrational ladder of the discretized continuum. To speed up the calculations, only some of the electronic states from the entire spectroscopic model are included, where couplings with the target state are non-negligible. For the A ^2^Δ state, we include X ^2^Π and b ^4^Π continua, while for the C ^2^Σ^+^ state we include X ^2^Π, B ^2^Σ^−^, and a ^4^Σ^−^ states with a large *v*_max_ ≥ 1000 in both cases. A limited spectroscopic model will change the energies of levels, but the resonance widths should stay largely unaffected by this, provided that the couplings with the omitted states are weak and that the energy shifts are not so large as to move levels below or above the respective dissociation limit.

### 2.3 Predissociation efficiency

Predissociation provides the system with an alternative route to de-excitation from the states above the dissociation threshold in addition to radiative decay; collisional effects and excited-state absorption are not considered in our model. The knowledge of radiative and predissociative lifetimes (or rates) enables calculating predissociation efficiency as

2 *η* = *k*_pred_/*k*_tot_,

where the total de-excitation rate is calculated as

3 *k*_tot_ = *k*_pred_ + *k*_rad_ = 1/*τ*_pred_ + 1/*τ*_rad_

The radiative lifetimes have been computed using ExoCross using a 13-state model described in our companion paper.^16^ Determination of the predissociation efficiency values is essential for the calculation of photodissociation cross-sections and rates; it is described in detail there.

## 3 Results and discussion

### 3.1 Predissociation in B ^2^Σ^−^ state

**Fig. 3:**
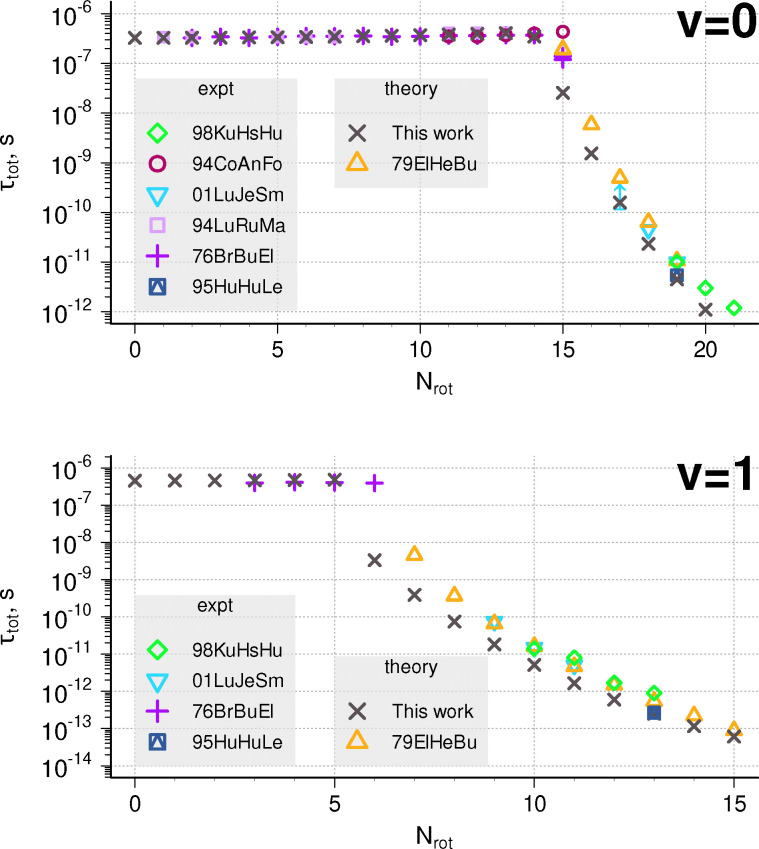
Comparison of lifetimes with available data (94CoAnFo,^37^ 94LuRuMa,^38^ 98KuHsHu,^20^ 01LuJeSm,^39^ 76BrBuEl,^19^ 95HuHuLe^40^) for the B ^2^Σ^−^ state, *v* = 0 (top) and *v* = 1 (bottom). The arrow mark at *N* = 17 represents the lower limit.

The B ^2^Σ^−^ state goes to the first dissociation limit, and its levels can predissociate only due to tunnelling through the PEC barrier. Fig. 3 compares our theoretical total lifetimes *τ*_tot_ for *v* = 0,1 with the literature data.^19,20,37–40^ Only the spin-independent *τ* value is plotted for the theoretical data, calculated using LEVEL with a single B ^2^Σ^−^ curve, without the account of *ρ*-splitting. In the experiment, Brzozowski *et al.*^19^ observed different behaviour for the two components of the *N* = 15 state, while Luque and Crosley^41^ observed no difference. The agreement between our data and the experiment is good for low *N* values, where only radiative lifetime matters. For high *N* values our theoretical lifetimes are too short, especially in the *v* = 1 state.

The largest disagreement is with the theoretical data by Elander *et al.*^23^ The accuracy of Weyl theory used by Elander *et al.*^23^ to obtain resonance widths should be within 10% of the semiclassical WKB-type approaches that LEVEL uses, so the discrepancy must come from the shape of the PEC. Indeed, a smaller barrier would make tunnelling more likely and reduce the resulting predissociation lifetimes, and a larger barrier would reduce the disagreement with Elander *et al.*^23^ However, our current barrier height of 670 cm^−1^ is large enough to obtain the quasi-bound upper state for the B–X (0-0) R1(21.5) transition, which was observed by Masseron *et al.*^6^ Elander *et al.*^23^ summarizes previous *ab initio* studies, and all the predictions for the barrier height fall into the range 500–1000 cm^−1^, similar to the curve used here.

We should note that the cause of the potential barrier is not entirely clear. Usually, such features appear as a result of avoided crossings, see Yurchenko *et al.*^42^ for example. However, the closest CH state with the same ^2^Σ^−^ symmetry is a 3pπ Rydberg state located ∼6 eV above it.^43,44^ Kalemos *et al.*^45^ say that based on population analysis, the barrier is due to the contribution from the ^3^D excited state of carbon, the dissociation energy into which is 8.6 eV above the first dissociation limit of CH producing ground-state atoms. Lie *et al.*^46^ also mention that ^3^D state, but point out that only the ionic configurations (*i.e.*, C^−^ + H^+^) that lie more than ∼14 eV above the CH dissociation energy could show an attractive enough character to cross the low-lying B ^2^Σ^−^ state.

It is possible that optimization of Rydberg orbitals^47^ at the MCSCF step or fitting of the potential^23^ to new experimental data could improve the agreement for predissociative and total lifetime predictions of the B ^2^Σ^−^ state. Fortunately for our use case, such a difference is not critical for the estimation of *η* as the radiative lifetimes for B ^2^Σ^−^ are orders of magnitude longer than *τ*_pred_, *τ*_rad_ > 500 ns for *v* = 0 and 529 ns for *v* = 1. Thus, as we go beyond the dissociation threshold, predissociation efficiency quickly approaches unity, *η* ≈ 1. So, for case III process in the B ^2^Σ^−^ state it is more important to accurately determine the onset of predissociation and get lifetimes for the first few levels, rather than the lifetimes of levels towards the top of the potential energy barrier.

Experimentally, the onset of predissociation is observed in low-pressure emission spectra as a drop in intensity within a branch with increasing *N* quantum number.^48^ Emission spectra are much more sensitive to predissociation and exhibit these effects well before the absorption lines start to noticeably broaden. Herzberg and Johns^48^ report the last observed and the first missing emission lines in the B–X (0,0) band at *N* = 15 and 16. Brooks and Smith^49^ using a phase-shift measurement setup observe a sharp lifetime reduction for *v* = 0, *N* = 15, and Brzozowski *et al.*^19^ observe intensity reduction in emission spectra going from both spin components of this level. Luque and Crosley^41^ determine the predissociation onset to be between *N* = 14 and 15 for *v* = 0. These data align reasonably well with our calculated values of *η* for *N* = 14, 15, 16, *η* = 0.17, 0.94, 0.997. The WKB approach should be very accurate for narrow, long-lived levels,^33,50^ so if the width of the barrier is approximately correct, we can still retrieve the predissociation threshold. Similarly, for transitions to *v* = 1, the line with *N* = 7 disappears from experimental emission spectrum,^48^ and the threshold is determined as *N* = 7^49^ or between *N* = 6 and 7,^41^ which reasonably compare with our predictions for *N* = 5, 6, 7, *η* = 0, 0.993, 0.999.

We therefore conclude that the use of the WKB approach for B ^2^Σ^−^ predicts the onset of predissociation with Δ*N* = ±1 accuracy and gives a reasonable description of rotational predissociation effects for long-lived states despite possible shortcomings of the *ab initio* calculations, the absence of fitting or couplings in calculations done with LEVEL. Only four levels belonging to *v* = 0, *N* = 14, 15 predissociate incompletely and contribute partially to photodissociation cross sections, whereas transitions to more excited states always leads to dissociation.

### 3.2 Predissociation in A ^2^Δ state

There has been a long history of discussions whether the A ^2^Δ state predissociates (see Carozza and Anderson^51^ and references therein), how efficient^19,53^ this process is, and what the consequences are for observations.^6^ To model predissociation for the A ^2^Δ state, we employ the stabilization procedure in addition to calculations of shape resonances, as the PEC correlates to the second dissociation limit and both predissociation cases I and III are possible. The A ^2^Δ PEC has a deep well whose minimum is below the first dissociation limit, but energetically dissociation is possible for all levels with *v* ≥ 2 and for some levels in *v* = 0,1 states.

**Fig. 4:**
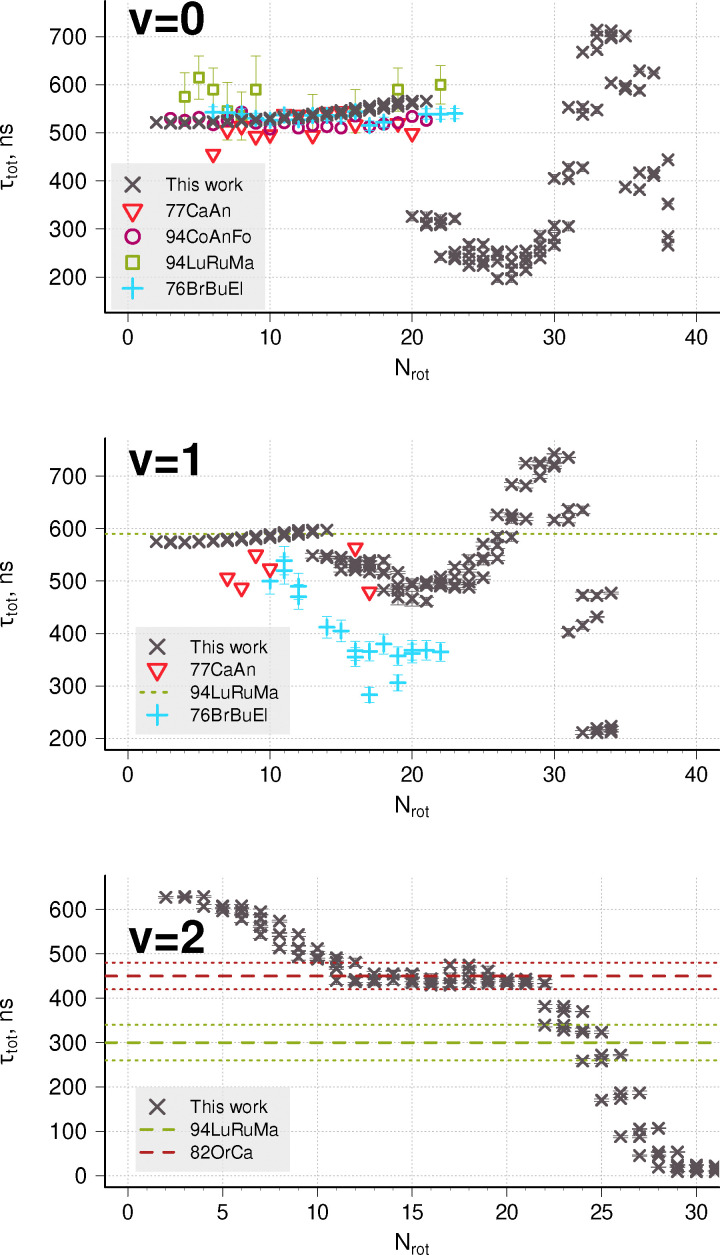
Comparison of total lifetimes with available data (77CaAnxx,^51^ 94CoAnFo,^37^ 94LuRuMa,^38^ 76BrBuEl,^19^ 82OrCa^52^) for the A ^2^Δ state, *v* = 0 (top) and *v* = 1 (middle) and *v* = 2 (bottom). Theoretical error bars represent 3*σ* of the Lorentzian fitting from the stabilization method. This does not include errors in LEVEL or the effect of uncertainties in PECs and couplings. Horizontal lines represent average values from Q-branch measurements with their respective error bars.

Predissociation in the lowest vibrational level has not been directly observed, and so is expected to be very weak. Fig. 4 compares the available lifetimes data with our calculations; all four levels due to spin–orbit splitting and *Λ*-doubling for each *N* are plotted for the theoretical data. The agreement for low *N*-values is good, but no drop in lifetimes has been experimentally observed in *v* = 0 up to *N* = 23.^19,38^ The results of our stabilization method predict the onset of predissociation at smaller values of *N*, already starting for some levels with *N* = 19 with rate *k*_pred_ ∼ 10^6^ s^−1^ and noticeably reducing lifetimes at *N* = 20. Luque and Crosley^54^ used existing experimental *τ*_expt_ and theoretical *τ*_rad_ data to estimate the onset at *N* = 18 with rates *k*_pred_ ∼ 10^5^ s^−1^ for levels up to *N* = 23.

The middle panel of Fig. 4 shows a comparison of total lifetimes with the available experimental data for *v* = 1. The horizontal bar is *N*-averaged value obtained by fitting a single exponential to the signal decay of the entire Q-branch. Here, the situation is reversed, and our lifetime predictions are longer than the state-resolved experiment and the onset shifted to higher *N*. The agreement is overall reasonable. A similar measurement in the (2-2) band is given by Luque *et al.*,^38^ Ortiz and Campos^52^ (bottom panel). For this vibrational state, all levels predissociate, and our calculated lifetimes are distributed around the reported mean values *τ* = 300 ± 40 and *τ* = 420 ± 30 ns.

**Fig. 5:**
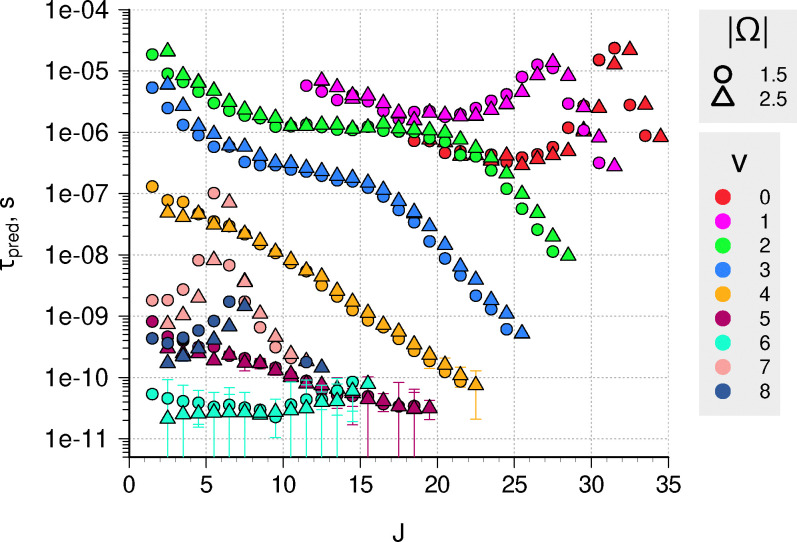
Calculated *τ*_case I_ predissociation lifetimes using stabilization method for various rovibronic levels of the A ^2^Δ state. Error bars represent one standard error, and for the long-lived states are too small to be seen.

Predissociation lifetimes for the A ^2^Δ rovibrational levels between the first and second dissociation thresholds are presented in Fig. 5. Our approach predicts a broad range of lifetimes, spanning six orders of magnitude from 20 µs to 20 ps. It is worth noting, that 20 µs is close to the limitation of this method: such long lifetimes correspond to resonance widths of about 10^−7^ cm^−1^. We had to modify DUO's output format to get more than six decimal places (the default ExoMol format) to obtain such narrow energy distributions. For the levels in the A-state, this corresponds to 13 significant digits, and numerical noise starts to be an issue.

The calculated lifetimes of A ^2^Δ show strong variation with both *J* and *v* quantum numbers, which can be qualitatively explained based on the underlying couplings and a two-state approximation for predissociation lifetimes*τ*_pred_ = 1/*k*_pred_ ∝ 1/|〈*ψ*^0^_cont_(*E*)|*H*_12_|*ψ*^0^_bound_(*E*)〉|^2^,where *H*_12_ is the coupling operator and *ψ*^0^ are the uncoupled wavefunctions. Just above the dissociation threshold, both spin components of ^2^Δ experience coupling to the continuum of the X ^2^Π state *via* the *Ĵ*_±_*L̂*_∓_ operator (L-uncoupling^55,56^). In DUO, this coupling is modelled *via* the *L*_*x*_ coupling curve, which for X and A states has a maximum around *r*_e_. This behaviour would cause the matrix element from the equation above to be reduced as *L*_*x*_(*r*) decreases at longer bond lengths, so the lifetimes of more excited levels become longer. In Fig. 5 this shows as an increase in *τ*_pred_ with *J* for *v* = 0 and 1, an increase from *v* = 0 to *v* = 1, and flat sections in *v* = 2 and 3. At around 37 000 cm^−1^ (*v* = 5–6), the A ^2^Δ curve is crossed by b ^4^Π to which it is coupled by spin–orbit interaction (≈10 cm^−1^) that again affects all spin states and causes a significant reduction of lifetimes in *v* > 4 and high *J* levels of lower vibrational states. Additionally, the X–A spin–orbit coupling only affects |*Ω*| = 1.5 (Herzberg case *d*^57^) levels, causing additional patterns in our predictions. Such a difference in lifetimes between different spin components has been observed before for CH.^19^ This explanation does not require invoking the weak coupling between B ^2^Σ^−^–A ^2^Δ^19^ as the main reason for predissociation; this is in line with analysis by van Dishoeck.^53^

It should be noted, that these lifetimes are obtained by fitting to a symmetric, Lorentzian distribution, which somewhat constrains the fitting procedure. For most states, this works quite well and the ratio between the standard error of the retrieved width and its magnitude is rather small, *σ*_*γ*_/*γ* < 0.05. However, for a number of states in the vicinity of the curve crossing between A ^2^Δ and b ^4^Π states (*v* = 5,6, and high *J* states with *v* = 4) the two values *γ* and *σ*_*γ*_ become comparable, indicating a poorer fit (see the error bars in Fig. 5). Relaxing the symmetric profile restriction and using asymmetric line-shapes, which are known in preionization and predissociation spectra^58,59^ and are usually described using the Fano profile,^60,61^ could in principle improve the retrieval accuracy for those states. This was done in the study of the Schumann–Runge band in O_2_, where the largest discrepancy between Lorentzian and Fano shapes was observed for the broadest resonances,^62^ similar to our findings here. However, due to the statistical nature of our approach and low number of counts in the wings of each resonance, any asymmetry, if present, would be hard to describe with any degree of confidence. Additionally, even if we refrain from quantum number filtering when building resonance histograms, the density of unbound states is still relatively low to observe the characteristic dip in the profile. Another issue affecting the fitting accuracy is the choice of the number of bins in relation to the binning window along the energy axis where we look for a resonance. We believe this aspect to be more important to the accuracy of the retrieved widths and lifetimes than asymmetry, but it has not been investigated in great detail; large uncertainties occur only for states with short lifetimes, where predissociation is already much faster than radiative decay and *η* ≈ 1.

**Fig. 6:**
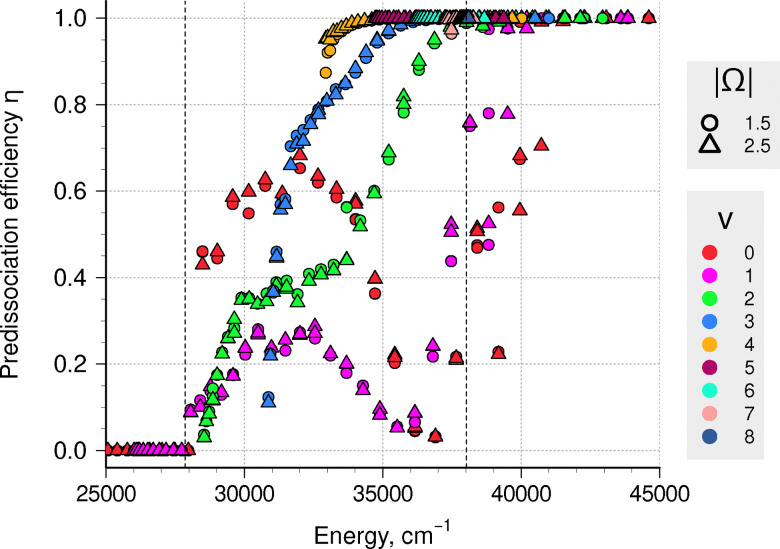
Calculated predissociation efficiency values for levels in the A ^2^Δ state. Vertical dashed lines mark the first and second CH dissociation limits, *D*_0_.

The case I predissociation lifetimes calculated using stabilization were augmented by case III WKB results to get total predissociation lifetimes and predissociation efficiencies; the latter are shown in Fig. 6. Radiative lifetimes for A ^2^Δ levels are of the order 0.5–1 µs, so a number of slowly predissociating levels in *v* = 0–3 experience competition between radiative and predissociative relaxation mechanisms. Levels with *v* ≥ 5 are expected to completely dissociate.

Here, it is interesting to note that, in addition to laboratory measurements, CH has been observed and identified in several high-resolution stellar spectra, and in those data the A–X transitions up to *v* = 5 do not show any increase in broadening^6^ that could be expected from a strong lifetime reduction. This, however, does not contradict our results as the Doppler broadening at equivalent temperatures of those stars (5000 K) in the frequency range of the A–X bands are *γ*_Dopp_ > 0.15 cm^−1^. The inclusion of natural linewidths would change the line profile to have the Voigt shape, but even for the shortest-lived states in *v* = 5 with *τ* = 30 ps, the resulting Voigt line halfwidths would increase only up to 0.20 cm^−1^. This is not enough to observe a noticeable broadening at those resolving powers. For discussions about the lack of intensity drop in stellar absorption spectra and the role of molecular predissociation, see Pavlenko *et al.*^63^ and Yurchenko *et al.*^64^

### 3.3 Predissociation in C ^2^Σ^+^ state

**Fig. 7:**
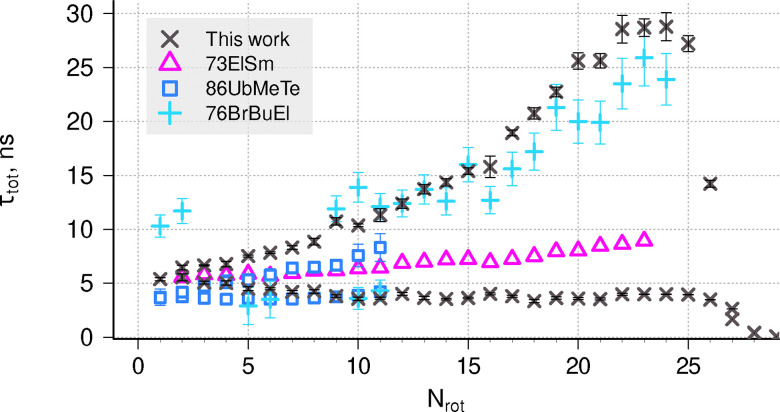
Comparison of total lifetimes for the C ^2^Σ^+^, *v* = 0 state with experimental data (73ElSm,^65^ 86UbMeMe,^66^ and 76BrBuEl^19^). Only the short-lived component of Elander and Smith^65^ is plotted. The data from Brzozowski *et al.*^19^ includes the contribution they attributed to *v* = 1.

Several experimental papers have been concerned with predissociation effects in the C–X (0,0) band.^19,48,65–68^ In a recent study, Ubachs *et al.*^66^ performed an accurate laser-induced fluorescence study and showed two distinct trends in experimental lifetimes for levels of different rotational symmetry e,f (*F*_1_ and *F*_2_), effectively resolving the issue of two different behaviours of lifetimes that has been contested in the past. Fig. 7 shows that the lifetimes for the e states are relatively constant at 3.7 ns, while for the f states they increase linearly from 3.7 ns at *N* = 1 to 8 ns at *N* = 11. Brzozowski *et al.*^19^ observed no short-lived component in *v* = 0 state, and instead attribute these short lifetime measurements *τ* ≈ 3 ns to *v* = 1 state (which, based on our calculations, would also have similar lifetimes). Our data for *v* = 0 (black crosses) reproduce the different behaviour for e and f levels and show best agreement with the data of Brzozowski *et al.*^19^ if their *v* = 1 data is treated as measurements of *v* = 0, e levels.

Other absorption measurements in flames^69^ and in plasma^68^ also targeted higher vibrational states by looking at (1,1) and (2,2) bands. However, their instrumental and Doppler linewidths were too large to observe any broadening due to predissociation, so only lower limits on the lifetimes can be deduced. Our *τ*_tot_ predictions for *v* = 1,2 are shorter than those for *v* = 0 but still in the nanosecond range, and agree with the suggested lower limits of 100 ps for *v* = 1 and 50 ps for *v* = 2.^68^

**Fig. 8:**
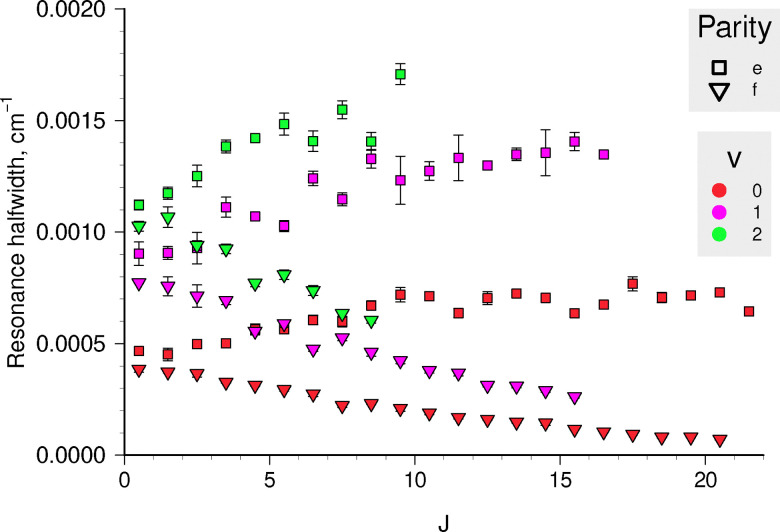
Calculated case I resonance widths for the C ^2^Σ^+^ rovibrational levels from stabilization method histograms.

As for the predissociation mechanism, several tentative explanations have been proposed to explain the difference between e and f levels' lifetimes, as well as the pseudo-independence of *τ* on the *N* quantum number for the e levels.^48,53,66^ The most probable candidate mechanisms are the C ^2^Σ^+^–B ^2^Σ^−^ spin–orbit, C ^2^Σ^+^–B ^2^Σ^−^ spin-rotation, and C ^2^Σ^+^–X ^2^Π rotational nuclear couplings. The experimental lifetimes of the e levels might appear pseudo-independent of *J* (or *N*),^66^ but according to our calculations that extend to higher *J* values, the underlying resonance widths, shown in Fig. 8, exhibit approximately linear dependence in opposite directions with slightly different magnitudes for the e and f components. Such a splitting pattern that is linear with *J* is in line with spin-rotation mechanism. However, our model does not include the **H**^SR^ term in the Hamiltonian, and this behaviour can alternatively be explained as a second-order coupling of rotational and electronic motions of the molecule in **H**^SO^ + **H**^ROT^, or more specifically to *J*^±^*L*^∓^ and *L*^±^*S*^∓^ operators.^55,70^ The same mechanism was successfully used to explain the linewidth differences of fine-structure components in the B ^3^Σ_u_^−^ state of O_2_ due to interaction with 1 ^3^Π_u_ state.^71^

Because the first-order effect *via***H**^SR^ is not included in the model, the difference in predicted behaviours of the e and f components has to be attributed entirely to the second-order effect, which is expected to be the dominant term.^55,66^ We believe, this effect primarily arises due to the X ^2^Π–C ^2^Σ^+^ interaction included in our calculations *via L*_*x*_ and *H*_SO_ curves. The DUO code automatically accounts for the coupling terms in **H**^ROT^ + **H**^SO^ once the respective curves are included in the spectroscopic model. A number of couplings among X ^2^Π, B ^2^Σ^−^, C ^2^Σ^+^, and a ^4^Σ^−^ states that affect both spin components equally were also present in the calculation.

**Fig. 9:**
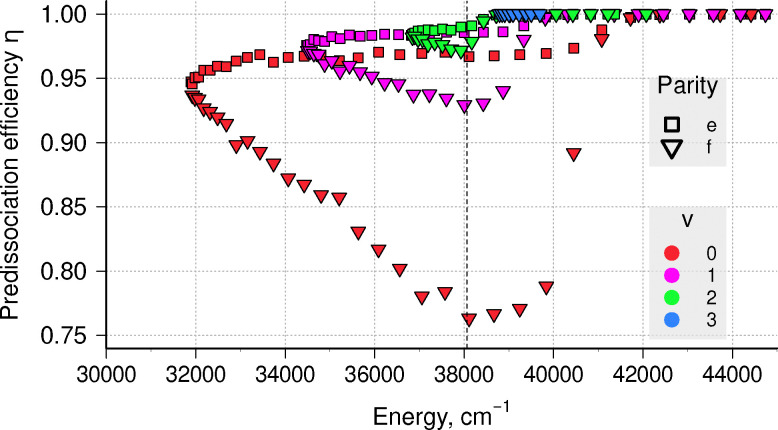
Calculated predissociation efficiency for levels in the C ^2^Σ^+^ state. Vertical dashed line is the second dissociation limit *D*_0_ of CH.

Radiative lifetimes in the C ^2^Σ^+^ state exceed 100 ns and slowly increase with *N* and *v*, but do not depend on parity. Thus, predissociation efficiency differs for e and f states, but remains high in both cases, as shown in Fig. 9. Only for the long-lived component in *v* = 0 does it go below 90%. For these *ab initio* curves, both DUO and LEVEL predict the existence of another *v* = 3 state above dissociation, where we set *η* = 1 due to small *τ*_case III_ lifetime value. This figure also illustrates why the observed intensity of emission lines originating from the two spin components is different, as the population of levels that do not dissociate is proportional to 1 − *η*, so emission from f-levels will be several times more intense.^48,65,67^

## Conclusions

In this paper, we present theoretical analysis of predissociation lifetimes for three excited electronic states of CH, A ^2^Δ, B ^2^Σ^−^, C ^2^Σ^+^ and compare them to the much simpler to compute^72^ radiative lifetimes. Our new implementation of the stabilization method was applied to the results of eigenenergy calculations using the DUO code in order to obtain *τ*_pred_ values for 628 quasi-bound levels in A ^2^Δ and 95 levels in C ^2^Σ^+^ states in the energy range between the first and second dissociation thresholds of CH. Additionally, for levels above their respective dissociation thresholds, predissociation lifetimes due to tunnelling were computed in the WKB approximation using the code LEVEL.

The comparison of total lifetimes, which includes both predissociation mechanisms and radiative decay, with the experimental data gives reasonable results for the C ^2^Σ^+^ and A ^2^Δ states. For the A ^2^Δ state, even below its dissociation threshold the lifetimes span six orders of magnitude and show a strong dependence on both *N* and *v* quantum numbers, while for the C ^2^Σ^+^ state the dependence on *v* and *N* is much weaker, and the experimentally observed difference in lifetimes of the e,f parities is reproduced. For both A ^2^Δ and C ^2^Σ^+^ states, predictions are obtained for all the allowed energy levels, including those difficult to reach experimentally, and predissociation mechanisms are proposed that explained the behaviour of our theoretical predictions. For the B ^2^Σ^−^ state, our *τ*_pred_ calculations are underestimated above the dissociation threshold, but the threshold itself is still accurate to within Δ*N* = ±1. However, this underestimation of *τ*_pred_ does not substantially alter *η*, the predissociation efficiency, for these states.

While in absorption studies the account of strong predissociative effects leading to line broadening is essential for modelling the observed spectra,^73^ weak predissociation considered in this work has a more subtle effect. When we compare the combined case I and III predissociation lifetimes *τ*_pred_ with radiative lifetimes, we show that for many levels, especially in the A ^2^Δ state, the two have a comparable magnitude, so in the absence of collisions, the molecules in those states are as likely to emit a photon as they are to dissociate. The knowledge of accurate *η* values for each individual state calculated in this work enables one to account for population effects and model temperature-dependent photodissociation cross-sections and photodissociation rates.^16^

Future work might include using a set of refined electronic structure data based on experimental transition frequencies^74^ for CH. Studies of other systems (notably H_2_) will need to account for additional dissociation mechanisms, such as radiative dissociation,^75^ for which the destination of any emitted photon must be explicitly considered. The present methodology should also be applicable to this process, but this mechanism is not a significant contributor to CH photodissociation. The stabilization approach methodology can also be improved so that it does not have to rely on approximate quantum number assignments and instead employs some sort of clustering algorithm to identify different levels within *J* and parity groups. This is likely to become necessary for the studies of molecules other than light hydrides for which the density of states increases.

## Author contributions

Andrei Sokolov: conceptualization, software, formal analysis, writing – original draft. Sergei N. Yurchenko: conceptualization, software, writing – review & editing. Jonathan Tennyson: conceptualization, writing – review & editing, funding acquisition.

## Conflicts of interest

There are no conflicts to declare.

## Supplementary Material

CP-028-D5CP04531B-s001

## Data Availability

Supplementary information: sample input files for DUO used for one step of stabilization calculations for A ^2^Δ and C ^2^Σ^+^ states; input files for LEVEL for all three states. See DOI: https://doi.org/10.1039/d5cp04531b. The supplementary information (SI) contain sample DUO input files used for stabilization calculations of A ^2^Δ and C ^2^Σ^+^ states and input files used with LEVEL. Images of all stabilization resonances and an ExoMol .states file containing total *ab initio* lifetimes and predissociation efficiencies (columns 5 and 13) are available in Zenodo (https://doi.org/10.5281/zenodo.17611612).
